# Impact of a bioethics and humanities program on the educational training of nephrology residents

**DOI:** 10.1093/ckj/sfaf298

**Published:** 2025-09-24

**Authors:** Guillermo Cantú Quintanilla, Irma Gómez Guerrero, Gloria Garcia-Villalobos, Geovana Martin Alemañy, Nuria Aguiñaga Chiñas, Rafael Valdez-Ortiz

**Affiliations:** School of Health Sciences, Universidad Panamericana, Mexico City, Mexico; Nephrology Department, Hospital General Dr Eduardo Liceaga, Mexico City, Mexico; Nephrology Department, Hospital General Dr Eduardo Liceaga, Mexico City, Mexico; School of Nutritional Sciences and Wellness, The University of Arizona, Tucson, AZ, USA; School of Health Sciences, Universidad Panamericana, Mexico City, Mexico; Nephrology Department, Hospital General Dr Eduardo Liceaga, Mexico City, Mexico

**Keywords:** humanistic and bioethical education, Mexico, nephrology residents

## Abstract

**Background:**

Modern medical training must integrate not only clinical skills but also ethical and humanistic competencies. In 2013, a structured program in bioethics and humanism was implemented as part of a nephrology residency curriculum. The objective of this study was to evaluate the impact of a 3-year humanism and bioethics program for nephrology residents that focused on improving clinical communication, reducing complaint and lawsuit numbers, increasing patient satisfaction, and supporting decision-making centered on quality of life.

**Methods:**

A longitudinal, ambispective cohort (2010–19), our 3-year curriculum delivered weekly 1-h sessions for 6 months/year to 45 residents and was facilitated by three faculty instructors across six core themes. To relate outcomes to the intervention, analyses were anchored to the 2013 launch and compared pre-program (2010–13) versus post-program (2014–19) rates of formal complaints, legal claims, patient satisfaction and maximum benefit discharges.

**Results:**

Formal complaints decreased from 47.8 to 26.0 per year [incidence rate ratio (IRR) 0.54, 95% confidence interval (CI) 0.44–0.67; *P* < .001; Holm <0.001]. Legal claims were reduced from 4.25 to 0.17 per year (IRR 0.039, 95% CI 0.005–0.295; *P* = .0016; Holm = 0.0016). Maximum benefit discharges increased from 4.25 to 76.5 per year (IRR 18.0, 95% CI 11.09–29.21; *P* < .001; Holm <0.001). For satisfaction, the ordinal logistic model showed an odds ratio (OR) of 3.53 (95% CI 1.96–6.38; *P* < .001; Holm = 0.0001), consistent with the dichotomous sensitivity analysis (≥4 vs ≤3) (OR 4.08, 95% CI 2.16–7.71; *P* < .000).

**Conclusions:**

The humanism and bioethics program was proven to be an effective and transformative educational tool that promoted ethical, empathetic and patient-centered nephrology practices. The positive impact of this program was evident in both clinical indicators and strengthened medical professionalism.

KEY LEARNING POINTS **What was known:**

- Very few articles in nephrology journals focus on structured humanistic education with clinical impact assessment.

**This study adds:**

- This is not just an anecdotal or testimonial course, but a longitudinal program with objective outcomes (complaints, demands, satisfaction, clinical decisions), which is rare in the field.

**Potential impact:**

- In a post-pandemic medical world where burnout, difficult decisions, palliative care and healthcare justice are discussed, your article proposes a structured educational strategy that improves not only skills but also the way medicine is practiced.

## INTRODUCTION

Humanistic education is an educational paradigm that focuses on the integral development of the human being beyond the acquisition of technical or specialized knowledge [1]. In the medical field, humanism is characterized by respectful and compassionate relationships between healthcare professionals and their patients [2]. Humanistic education aims to cultivate intellectual, emotional and social capacities by fostering critical thinking, creativity, empathy and sensitivity toward the person receiving care [3].

Medicine is not limited to scientific and technical knowledge. Humanistic education complements medical training by providing tools for understanding the social, cultural and psychological dimensions of health and disease [4]. Humanistic education promotes the strengthening of essential skills, such as effective communication, active listening, empathy, ethics and teamwork [5]. By placing the patient at the center of the physician–patient relationship, humanistic education recognizes the educational, cultural and social complexity and diversity of individuals. Thus, this educational approach aims to develop physicians who have greater cognitive, emotional, social and ethical capacity, enabling them to analyze information, question preconceived ideas and make informed decisions [6, 7].

The importance of strengthening the physician–patient relationship has been recognized for nearly a century [8]. In the late 1970s, Gorlin and Zucker developed a structured 4-year curriculum in humanistic medicine at the Department of Medicine of Mount Sinai School of Medicine [9]. This model sought to enhance communication skills, empathy and the physician–patient relationship. Although no objective evaluations were published, the authors reported improvements in attitudes and behaviors among third-year residents and graduates that aligned with a biopsychosocial approach to medical practice [10].

In 2010, the nephrology residency program was established at our hospital [11] and is accredited by the National Autonomous University of Mexico (UNAM, according to its Spanish acronym) [12]. Since its inception, the residency has emphasized not only technical competence but also preparation to navigate one of the most complex specialties in medicine [13]. In 2013, we introduced a structured 3-year curriculum in humanism and bioethics, delivered in parallel to—and as an adjunct to—the standard nephrology academic program. This curriculum exposes residents to philosophical, ethical and clinical communication content to enrich their professional development. Our humanism–bioethics approach focuses on concrete competencies—communication, shared decision-making, values-based reasoning and professional practice *in situ*—rather than abstract terminology. The objective of this study was to characterize the program’s effect on care by comparing pre- versus post-implementation (2010–13 vs 2014–19) rates of formal complaints, legal claims, patient satisfaction (5-point Likert) and “maximum benefit” discharges at our institution.

## MATERIALS AND METHODS

### Study type

This was a cohort study that aimed to evaluate an educational program in bioethics and humanities applied to nephrology residents. The course, which was initially offered in 2013, begins each March with the arrival of new residents, and it includes 1-h sessions that are conducted weekly for 6 months. The program has continued uninterrupted to the present day. For this study, we analyzed the impact of the educational program from March 2013 to December 2019.

### Study design

A longitudinal, ambispective, prospective and observational study was conducted within the Nephrology Department to evaluate the impact of a humanism and bioethics program as part of the clinical and holistic medical training of nephrology residents.

### Educational setting and humanism–bioethics intervention

At the General Hospital of Mexico Dr Eduardo Liceaga, Nephrology is a 3-year subspecialty within UNAM’s Single Program of Medical Specialties (PUEM). Candidates enter after 2–4 years of Internal Medicine, having passed the national residency examination (ENARM) and the hospital’s admission process (Table 1). From 2010 to 2013, 10 residents completed nephrology training; from 2013 to 2019, 45 residents completed the program, with an average of 15 active residents per year (PGY = postgraduate year; PGY-1: 5; PGY-2: 5; PGY-3: 5). The humanism/bioethics curriculum ran in parallel to the academic program (weekly 1-h sessions, 6 months per year), facilitated by three faculty, with an average of 15 residents attending per year. For the 3-year curriculum (Table 2), in the first year, the sessions covered the fundamentals of philosophical anthropology: sensitive life, intellect and emotions, the human person, technology, science and values, freedom, interpersonal relationships, happiness and the meaning of life, social life, sexuality and the family, law and justice, culture, economy, politics, time, human limitations, destiny and transcendence. The second year included studies on knowledge of philosophy, science, logic and truth; human reality; scientific and philosophical worldviews; biology, nature and culture; human actions, including labor, technical progress, artistic creation, and ethics and moral systems; and societal structures, such as the family, justice, law, politics and the state. The third year focused on current issues in bioethics that were chosen by residents and concluded with a communication workshop about how to deliver bad news.

**Table 1:** Overview of residency structure and attendance—nephrology (UNAM–PUEM), General Hospital of Mexico Dr Eduardo Liceaga.

Component	Details
Setting	General Hospital of Mexico “Dr Eduardo Liceaga.” Nephrology is a 3‑year subspecialty within UNAM’s Single Program of Medical Specialties (PUEM)
Entry requirements	2–4 years of Internal Medicine, successful completion of the national residency examination (ENARM) and successful completion of the hospital’s selection process
Resident completions (2010–13)	10 residents completed nephrology training
Resident completions (2013–19)	45 residents completed the program
Active residents per year	An average of 15 active residents per year (PGY‑1: 5; PGY‑2: 5; PGY‑3: 5)
Humanism/bioethics curriculum	Delivered in parallel to the academic program: weekly 1‑h sessions, 6 months per year; facilitated by 3 faculty
Average attendance (curriculum)	An average of 15 residents attending per year
PUEM academic modules (March–December; on‑site 1‑h classes, Mon–Fri)	1. Renal Physiology
	2. Acid–Base Disorders
	3. Fluid and Electrolyte Disorders
	4. Diabetes Mellitus
	5. Hypertension
	6. Glomerular Diseases
	7. Acute Kidney Injury
	8. Chronic Kidney Disease
	9. Nephrogeriatrics
	10. Onconephrology
	11. Kidney Transplantation
	12. Obstetric Nephrology
	13. Critical Care Nephrology
	14. Miscellaneous
Clinical rotations (residents)	1. Inpatient (Hospitalization)
	2. Peritoneal Dialysis Clinic
	3. Ambulatory Hemodialysis Unit
	4. Interventional Nephrology
	5. Kidney Transplantation
	6. Critical Care Nephrology
	7. Glomerulopathies & Renal Pathology
	8. Outpatient Clinic
Resident evaluations (annual)	1. Research Seminar and Thesis
	2. Academic Seminar
	3. Clinical Care Work
	4. Modular Examinations
Faculty (PUEM academic program)	1 course director, 2 assistant professors and 8 associate professors

**Table 2:** Educational program in humanism and bioethics.

Ethics and humanism	Humanities in medicine	Bioethics
1. Philosophical anthropology	1. The ideal of human excellence	1. Science and controversies
2. Human faculties	2. The person	2. Contemporary schools of thought
3. Key areas for personal development	3. Technology and the human world	3. Normative foundations
4. Fundamental ethical duties	4. Science, values and truth	4. Human corporality
5. The trilogy of human behavior	5. Freedom	5. Human sexuality
6. Ethical principles of conduct	6. Interpersonal relationships	6. Politics and law
7. Law and moral conscience	7. Sexuality and family	7. The human genome
8. Purpose-driven human action	8. Social life	8. Scientific status of the embryo
9. Ethical self-improvement	9. Happiness and meaning in life	9. Assisted reproduction techniques
10. Ethical implications of responsibility	10. Life	10. Prenatal eugenics
11. Principles of ethical conduct	11. Culture	11. Stem cells and cloning
12. Virtues in the workplace	12. Economic life	12. Abortion and contraception
13. Virtues in coexistence	13. Time and the human life cycle	13. HIV/AIDS
14. Teamwork	14. The city and political life	14. Brain and bioethics
15. Personal growth through work	15. Human limits	15. Brain death
16. Family and society	16. Destiny and religion	16. Euthanasia and assisted suicide
17. Human sociability	17. Minimal philosophy	17. Palliative care
18. Human love	18. The utility of philosophy	18. Breaking bad news in nephrology
19. Education and manipulation	19. Limits of science	19. Verbal and nonverbal communication
20. Authority in society	20. Fallacies and sophisms	20. SPIKES protocol*
21. Axiology	21. Truth criteria	21. Workshop on delivering bad news
22. Schools of value theory	22. Scientific worldviews	
23. Education in values	23. Metaphysical worldviews	
24. Hierarchy of values	24. Cosmos and life	
25. Harmony of values	25. Intelligence and language	
	26. Multiculturalism	
	27. Mind–body dualism	
	28. Work and technical progress	
	29. Artistic creation	
	30. Ethical behavior	
	31. Ethical systems	
	32. Justice and the law	
	33. Politics and the state	

* SPIKES protocol: setting, perception, invitation, knowledge, empathy, summary.

On the basis of this structure, the program was organized around six pedagogical pillars:

(i) Phenomenology, which promotes a stance of peaceful coexistence grounded in a lucid understanding of reality. This perspective fosters serenity and self-awareness, which are fundamental for meaningful professional interactions [14–16].
(ii) Anthropology, which emphasizes the formation of intelligence, will and character as inseparable components of human development. Intelligence is approached as dialogical knowledge, will as the force of self-determination and character as the regulation of emotions through reason [17–19].
(iii) Bioethics, which takes a first-person perspective as a reflection on human flourishing and moral self-governance. In contrast to contemporary relativism, the notion of a fulfilled life is reclaimed as the outcome of conscious ethical decisions that are made in dialogue with oneself and others [20, 21].
(iv) Ergology, in which medical work is viewed as a means of self-realization and service to others. A philosophy of work that is grounded in human virtues is proposed to enhance effectiveness and well-being in the workplace [22, 23].
(v) Sociability, which is centered on medical authority as a service to the autonomy and responsibility of others in healthcare contexts. Civic humanism empowers physicians as ethical agents within institutions and the broader community [24, 25].
(vi) Axiology, in which trains residents in values through an educational lens of resistance to massification, utilitarianism and meaninglessness. Inspired by Socratic philosophy, axiology encourages students to unlearn false knowledge to reframe medical practice with an existential orientation [26].

To facilitate replication, we provide a session-by-session syllabus with learning objectives (Supplementary data, Table S1 and S2). In the main text we use plain language (e.g. “values and practical reasoning,” “professional practice and work ethics”); precise definitions are provided in Supplementary data, Appendix A (A1).

### Statistical analysis and outcome variables

To evaluate the impact of the program between March 2013 and February 2020, four main outcome variables were analyzed: (i) the number of formal complaints received by the Quality and Patient Safety Unit of the Hospital General de México; (i) the number of legal claims (civil or criminal) filed against the Nephrology Department; (iii) the results of outpatient nephrology satisfaction surveys that were conducted during routine quality audits (anonymous surveys that used a five-point Likert scale ranging from “very dissatisfied” to “very satisfied” [27] and were administered by nursing staff unrelated to the humanism and bioethics program]; and (iv) the number of discharges designated “maximum benefit”—an institutional code applied to patients with low expected survival per the Charlson Comorbidity Index [28] documenting a shared decision by the patient, family and clinical team to pursue conservative kidney management rather than invasive renal replacement therapy. All variables are presented as numbers and proportions. Counting variables (formal complaints, legal claims and maximum benefit discharges) were compared between the pre-program (2010–13) and post-program (2014–19) periods using incidence rate ratios (IRR), assuming Poisson counts and using the period duration (years) as the exposure. We estimated 95% confidence intervals (CI) and *P*-values (Wald tests on the log IRR). To control Type I error across multiple endpoints, we applied the Holm–Bonferroni correction (α = 0.05). Theme-level analyses (complaints and legal claims). To assess whether the distribution of themes changed between periods, we constructed 5 × 2 contingency tables (theme × period) and applied Pearson’s χ² test of independence. Because some cells had small expected counts, we also ran per-theme 2 × 2 contrasts (each theme vs all other themes) using two-sided Fisher’s exact tests, reporting odds ratios (OR; post vs pre) with 95% CI when estimable; multiplicity across the five themes was controlled with Holm–Bonferroni (α=0.05). Satisfaction (5-point Likert) was analyzed with ordinal logistic regression (proportional-odds model), reporting OR with 95% CI; as a sensitivity analysis, we fitted a binary logistic model (≥4 vs ≤3). Analysis of variance was not used for binary or ordinal outcomes.

## RESULTS

### Resident cohort and training exposure

Data from the Nephrology Department and the Quality and Patient Safety Unit of the Hospital General de México were analyzed for 2010–19. In the pre-program period (2010–13), 10 residents completed nephrology training (mean age 32 ± 1 years; 5 women, 50%). After the program was introduced in 2013, 45 residents (2013–19) participated in the humanism/bioethics curriculum (mean age 31 ± 2 years; 23 women, 51%).

### Outcomes and linkage to the intervention

Outcomes were department-level (complaints, legal claims, satisfaction surveys and “maximum benefit” discharges) and thus not exclusively resident-dependent. Nevertheless, they are sensitive to resident-facing processes (communication, consent, shared decision-making and discharge planning). We therefore anchored analyses to the 2013 implementation and compared pre- vs post-program rates, interpreting results with appropriate caution.

### Formal complaints

**Figure 1:**
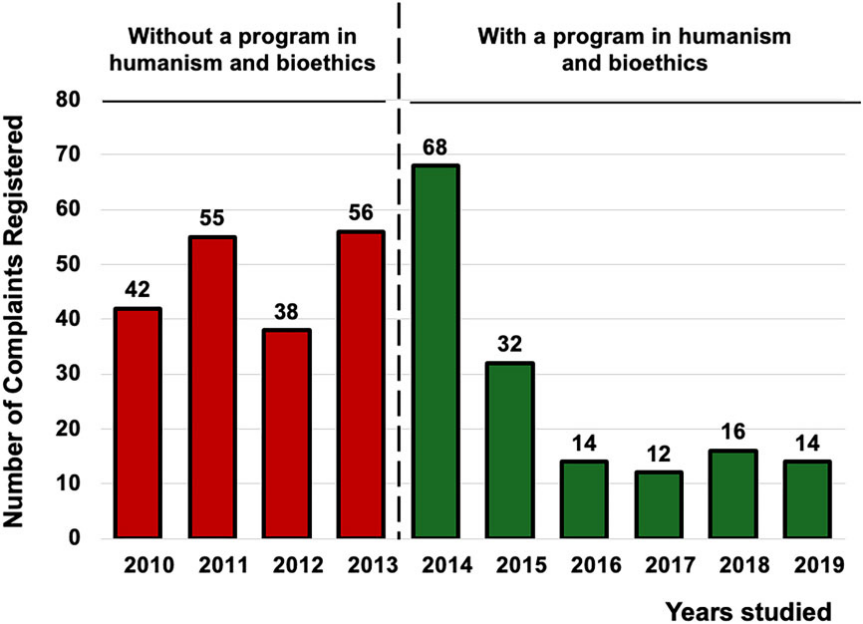
Number of formal complaints registered in the Nephrology Department and the Quality and Patient Safety Unit. Red bars represent the years during which the residents did not receive humanism and bioethics training. Starting in 2013, the green bars represent the years when the program was implemented. Formal complaints decreased from 47.8 to 26.0 per year with an IRR of 0.54 (95% CI 0.44–0.67; *P *< .001; Holm <0.001).

A total of 347 complaints were registered (2010–19): 191 pre-program (2010–13; 47.8/year) and 156 post-program (2014–19; 26.0/year). Rates declined (IRR = 0.54, 95% CI 0.44–0.67; *P* < .001; Holm <0.001; Fig. 1). Table 3A outlines the main themes of complaints to contextualize the observed reduction. Compared with 2010–13, the share of communication/information complaints decreased from 31% to 21% (*P* = .027). Attitude/respect complaints also declined substantially, from 40% to 12% (*P* < .001). In contrast, delays/waiting time increased from 6% to 43% (*P* < .001), and administrative/billing from 3% to 10% (*P* = .005). Clinical management/procedures showed a small, non-significant decrease (*P* = .256).

**Table 3:** Main issues in complaints and legal claims.

		Total	Pre	Post	
Complaint theme	Definition/example	*n* = 347 (%)	*n* = 191 (%)	*n* = 156 (%)	*P*-value
**(A)** Formal complaints—thematic breakdown					
Communication/information	Clarity of explanations, informed consent, expectations	92 (26)	60 (31)	32 (21)	.027
Delays/waiting time	Scheduling, clinic flow, dialysis chair availability	79 (23)	12(6)	67 (43)	<.001
Attitude/respect	Perceived disrespect, empathy, professionalism	94 (27)	76 (40)	18 (12)	<.001
Clinical management/procedures	Treatment decisions, procedural complications	61(18)	38 (20)	23 (15)	.256
Administrative/billing	Paperwork, authorizations, charges	21(6)	5 (3)	16 (10)	.005
		Total	Pre	Post	
Legal issue theme	Definition/example	*n* = 18 (%)	*n* = 17 (%)	*n* = 1 (%)	*P*-value
**(B)** Legal claims—thematic breakdown					
Informed consent	Alleged lack/defect in consent documentation	1 (6)	1 (6)	0 (0)	1.000
Diagnostic/treatment delay	Alleged delayed diagnosis or therapy initiation	6 (33)	6 (29)	0 (0)	1.000
Procedure‑related	Complications or technique‑related allegations	3 (17)	3 (18)	0 (0)	1.000
Professional conduct	Alleged negligence or malpractice	7 (38)	6 (35)	1 (100)	.411
Administrative	Documentation, referral, or authorization issues	1 (6)	1 (6)	0 (0)	1.000

### Legal claims

**Figure 2:**
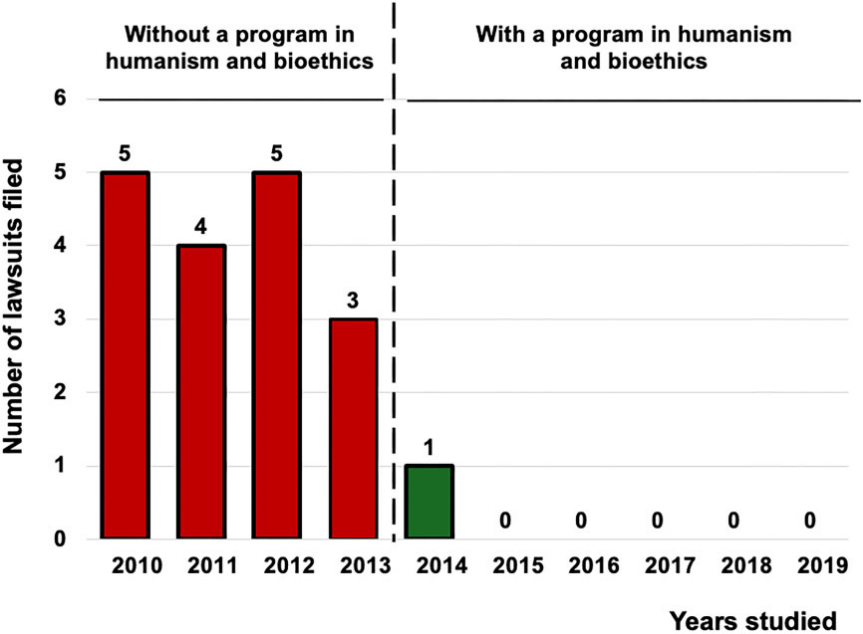
Number of legal claims filed against the Nephrology Department. Legal claims were reduced from 4.25 to 0.17 per year with an IRR of 0.039 (95% CI 0.005–0.295; *P *= .0016; Holm = 0.0016).

There were 18 legal claims overall (10 criminal, 8 civil). Most occurred pre-program (17/18 in 2010–2013). In per-theme, no category changed significantly (Table 3B). The principal signal remains the overall reduction in claims (IRR 0.039, 95% CI 0.005–0.295; *P* = .0016; see Fig. 2).

### Patient satisfaction

**Figure 3:**
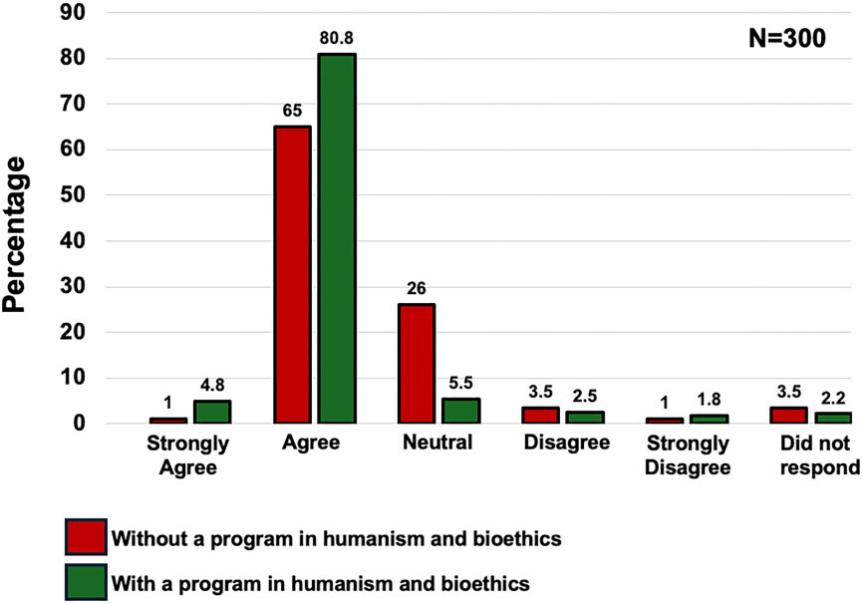
Patient satisfaction with nephrology outpatient consultations. Comparisons between patients treated by residents who did not receive humanistic training (red bars) and patients treated by residents who did receive humanistic training (green bars). The ordinal logistic model showed an OR of 3.53 (95% CI 1.96–6.38; *P *< .001; Holm = 0.0001), consistent with the dichotomous sensitivity analysis (≥4 vs ≤3) (OR 4.08, 95% CI 2.16–7.71; *P *< .000).

Outpatient surveys (≈30 per year, 2010–19; administered by the hospital’s Quality Unit) showed higher ratings in the post-program period. Ordinal logistic regression demonstrated greater odds of higher satisfaction categories post-implementation (OR 3.53, 95% CI 1.96–6.38; *P* < .001; Holm = 0.0001), consistent with a dichotomous sensitivity (≥4 vs ≤3: OR 4.08, 95% CI 2.16–7.71; *P* < .001; Fig. 3).

### Maximum benefit discharges

**Figure 4:**
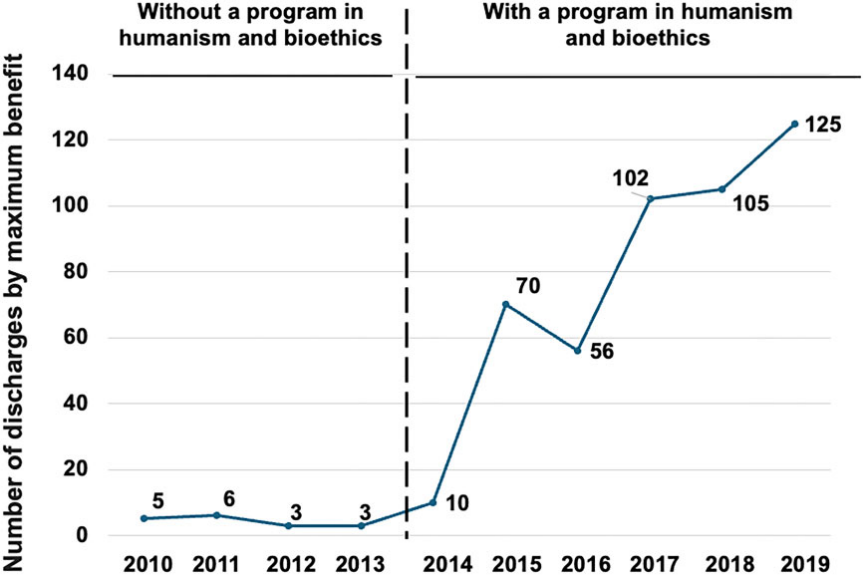
Maximum benefit discharges increased from 4.25 to 76.5 per year with an IRR of 18.0 (95% CI 11.09–29.21; *P *< .001; Holm <0.001).

Between 2010 and 2019, 476 patients were discharged under “maximum benefit” (shared decision for conservative management in patients with low expected survival per Charlson). Of these, 459 (96%) occurred in 2014–19. Rates increased (IRR 18.0, 95% CI 11.09–29.21; *P* < .001; Fig. 4).

## DISCUSSION

In an era where speed and technology dominate clinical environments, it is imperative to reconsider the humanistic dimension of medical practice. As a response to this need, the Nephrology Department at the Hospital General de México implemented a training program in the humanities targeted at nephrology residents with the aim of fostering a comprehensive view of both the patient and the healthcare professional. Inspired by contemporary philosophical practices that highlight the importance of formative concepts in medicine [6], the program was designed around a pedagogy that promotes the moral, social and professional development of physicians-in-training.

Turning to outcomes, implementation of the structured humanism and bioethics program was associated with sustained improvements in department-level indicators and patient experience. After the program’s launch, formal complaints declined from 47.8 to 26.0 per year (IRR 0.54), legal claims decreased from 4.25 to 0.17 per year (IRR 0.039) and “maximum benefit” discharges increased from 4.25 to 76.5 per year (IRR 18.0). In parallel, patient satisfaction—measured by an anonymous 5-point survey administered by the Quality Unit [27]—shifted toward higher categories (ordinal logistic OR 3.53) and showed a clinically relevant absolute increase (+21 percentage points). All effects remained statistically significant after multiplicity control and were directionally robust in sensitivity analyses.

The most plausible mechanism linking resident training to these changes is the curriculum’s emphasis on clinical communication, shared decision-making and explicit ethical deliberation (quality of life, dignity, autonomy) [6, 29]. This interpretation aligns with established frameworks that place shared decision-making at the core of patient-centered care and offer practical consultation models [30, 31], and with evidence that better physician–patient communication is associated with fewer malpractice claims [32]. Consistently, the thematic breakdown of complaints showed marked reductions in “communication/information” and “attitude/respect,” domains plausibly sensitive to resident–patient interaction and consent practices, whereas “delays/waiting time” increased—pointing to system-level bottlenecks (scheduling, clinic flow, dialysis access) largely beyond the scope of a training intervention. The rise in “maximum benefit” discharges coheres with more deliberate, patient-centered choices for individuals with limited expected survival (per Charlson) and with a documented, shared election of conservative management—behaviors the curriculum seeks to strengthen. Moreover, cultivating empathy and humanistic attitudes during training has been linked to better clinical outcomes in other settings, lending plausibility to the pathway from education to patient-facing metrics [33]. For legal claims, the overall rate dropped substantially; theme-specific comparisons were underpowered because only one claim occurred post-implementation, so granular causal inferences at the theme level are unwarranted.

Several limitations merit emphasis. The ambispective, non-randomized, single-center design without contemporaneous controls cannot exclude secular trends or other institutional changes. Outcomes are department-level rather than resident-exclusive, so they are best interpreted as proximal proxies likely influenced by resident-facing processes rather than as proof of direct causation. Modeling relied on annual rates with years as exposure; the absence of clinical denominators (e.g. visits, dialysis sessions) limited offset-based Poisson/negative-binomial and segmented interrupted time-series approaches. Satisfaction data were brief and lack full psychometrics; although the ordinal model is appropriate, the proportional-odds assumption could not be exhaustively tested and annual samples were modest [27]. Legal-theme analyses are underpowered due to sparse post-program counts (*n* = 1). Additionally, changes over time in reporting, coding or complaint-handling procedures could have influenced counts and could not be audited. Finally, external validity may be constrained by the public tertiary setting and the specific UNAM–PUEM residency context.

This initiative represents a concrete response to the growing dehumanization of medicine, integrating philosophical knowledge with clinical practice [29]. Training in the humanities is not an academic luxury but an urgent necessity for building a model of care centered on both patient and physician dignity. The six thematic pillars of the program enable residents to gradually become not only more competent specialists but also better individuals and citizens. Taken together with the observed reductions in complaints and legal claims, the upward shift in satisfaction and pattern-specific changes in complaint themes, these findings support the view that a systematic humanism/bioethics curriculum can translate into better communication, fewer medicolegal frictions, more prudent decision-making and a measurably improved patient experience.

## Supplementary Material

sfaf298_Supplemental_Files

## Data Availability

The data underlying this article are available in the article itself.
