# Supplementary material for: Impact of a bioethics and humanities program on the educational training of nephrology residents
Source: Clin Kidney J. 2025 Sep 24;18(10):sfaf298. doi: 10.1093/ckj/sfaf298 (PMC12538288; doi:10.1093/ckj/sfaf298)
Supplement: sfaf298_Supplemental_Files [file sfaf298_supplemental_files.zip › Supplement Tables S1 S2.docx]

# Supplementary Tables S1–S2

## Table S1. Three‑year syllabus (themes and exemplar sessions)

| Year | Themes / exemplar sessions | Learning objectives (examples) | Dose & delivery |
| --- | --- | --- | --- |
| Year 1 | Philosophical anthropology: sensitive life; intellect & emotions; personhood; technology, science & values; freedom; relationships; happiness & meaning; social life; sexuality & family; law & justice; culture/economy/politics; time/limits; destiny/transcendence. | Recognize personhood/dignity; relate values to clinical choices; identify ethical concerns in routine encounters; practice respectful communication. | Weekly 1‑h, in‑person; 6 months/year; seminars + short cases. |
| Year 2 | Knowledge & truth; logic/scientific method; human reality; scientific vs philosophical worldviews; biology, nature, culture; human actions (work, technical progress, artistic creation); ethics & moral systems; social structures (family, justice, law, politics, state). | Apply reasoning under uncertainty; integrate scientific & ethical judgments; analyze clinical policies with justice/equity lenses. | Weekly 1‑h; 6 months/year; seminar + case debate. |
| Year 3 | Contemporary bioethics chosen by residents (consent capacity, end‑of‑life, resource allocation, dialysis initiation/withdrawal); communication workshop on delivering bad news (SPIKES‑style). | Conduct shared decision‑making; lead family meetings; document goals of care; practice difficult‑conversation skills. | Weekly 1‑h; 6 months/year; seminars + skills workshop. |

## Table S2. Codebook for outcomes (themes and operational definitions

| Category | Operational definition / examples | Inclusion / exclusion rules | Mapping notes |
| --- | --- | --- | --- |
| Complaints — Communication / information | Clarity of explanations; information sharing; informed consent; expectation‑setting. | Include when primary issue is explanation/consent; exclude pure administrative delays. | Resident‑sensitive; cross‑link to consent documentation when applicable. |
| Complaints — Delays / waiting time | Scheduling; clinic flow; dialysis chair availability; prolonged waiting. | Include process/throughput issues; exclude attitude/communication unless primary. | System bottlenecks; non‑resident specific. |
| Complaints — Attitude / respect | Perceived disrespect; empathy; professionalism; tone. | Include demeanor/respect; exclude content‑focused communication failures. | Resident‑sensitive domain. |
| Complaints — Clinical management / procedures | Treatment decisions; procedural complications. | Include management choices or technique issues; tie to index encounter. | May overlap legal claims; keep primary driver here. |
| Complaints — Administrative / billing | Paperwork; authorizations; charges; referrals. | Administrative domain; exclude clinical/process issues. | Often external dependencies. |
| Legal — Informed consent | Alleged lack/defect in consent documentation. | Use hospital legal coding; link to chart forms if available. | Map to complaint 'communication' if co‑reported. |
| Legal — Diagnostic/treatment delay | Alleged delayed diagnosis or therapy initiation. | Document timestamps; exclude non‑clinical delays. | Process‑of‑care focus. |
| Legal — Procedure‑related | Technique/complication allegations. | Identify operator/setting; exclude consent‑only issues. | Link to management/procedure complaint if present. |
| Legal — Professional conduct | Alleged negligence or malpractice. | Generic legal bucket; assign specific theme when clear. | High‑level classification. |
| Legal — Administrative | Documentation/referral/authorization issues. | Non‑clinical legal matters. | Map to administrative complaint if present. |
| Maximum benefit (discharge) | Shared decision to pursue conservative kidney management in low expected survival (Charlson) with documented discussion (patient/family/clinical team). | Requires explicit note of shared decision and Charlson‑based prognosis. | Institutional code; capture date/service/sign‑off. |
