# Supplementary material for: Impact of a bioethics and humanities program on the educational training of nephrology residents
Source: Clin Kidney J. 2025 Sep 24;18(10):sfaf298. doi: 10.1093/ckj/sfaf298 (PMC12538288; doi:10.1093/ckj/sfaf298)
Supplement: sfaf298_Supplemental_Files [file sfaf298_supplemental_files.zip › Supplementary Appendix A.docx]

# Supplementary Appendix A — Replication Materials

This appendix provides operational details to facilitate independent replication of the humanism–bioethics curriculum and the analytic approach. Materials are organized as follows: conceptual definitions (A1); session-by-session syllabus (A2, Table S1); teaching materials and artefacts (A3); satisfaction instrument (A4); codebook for outcomes (A5, Table S2); fidelity and exposure (A6); analytic notes (A7); and a schematic/timeline (A8).

## A1. Conceptual definitions

**Humanism in medicine:** educational practices and attitudes that foreground dignity, empathy, respect, and holistic understanding of the person within clinical care.

**Bioethics:** structured ethical reasoning applied to clinical decision-making, including principles of autonomy, beneficence, nonmaleficence, and justice.

**Conservative kidney management:** planned, patient‑centred care for advanced CKD without dialysis, emphasizing symptom control and quality of life, aligned with patient values.

**Maximum benefit discharge:** institutional designation for patients with low expected survival per the Charlson Comorbidity Index, documenting a shared decision by the patient, family, and clinical team to pursue conservative kidney management rather than invasive RRT.

## A2. Session-by-session syllabus (overview)

Delivery: weekly 1‑hour, in‑person seminars during 6 months each academic year, over a 3‑year cycle (UNAM–PUEM nephrology residency). Faculty: 3 instructors. Average attendance: ~15 residents/year. The curriculum ran in parallel to the nephrology academic program (2013–2019).

**Table Appendix:** Three‑year syllabus (themes and exemplar sessions)

| Year | Themes / exemplar sessions | Learning objectives (examples) |
| --- | --- | --- |
| Year 1 | Philosophical anthropology fundamentals: sensitive life; intellect and emotions; the human person; technology, science and values; freedom; interpersonal relationships; happiness and meaning of life; social life; sexuality and family; law and justice; culture, economy, politics; time and human limitations; destiny and transcendence. | Recognize personhood and dignity; relate values to clinical choices; articulate ethical concerns in routine encounters; practice respectful communication. |
| Year 2 | Knowledge and truth; logic and scientific method; human reality; scientific and philosophical worldviews; biology, nature, culture; human actions (work, technical progress, artistic creation); ethics and moral systems; social structures (family, justice, law, politics, state). | Apply reasoning frameworks to uncertainty; integrate scientific and ethical judgments; analyze clinical policies with justice and equity lenses. |
| Year 3 | Contemporary bioethics selected by residents (e.g., consent capacity, end‑of‑life, resource allocation, dialysis initiation/withdrawal); communication workshop on delivering bad news. | Conduct shared decision‑making; lead family meetings; document goals of care; practice difficult‑conversation skills. |
